# Hierarchical Naive Bayes for genetic association studies

**DOI:** 10.1186/1471-2105-13-S14-S6

**Published:** 2012-09-07

**Authors:** Alberto Malovini, Nicola Barbarini, Riccardo Bellazzi, Francesca De Michelis

**Affiliations:** 1 Department of Industrial and Information Engineering, University of Pavia, Pavia, 27100, Italy; 2 IRCCS Fondazione Salvatore Maugeri, Pavia, 27100, Italy; 3 ICB, Weill Cornell Medical College, New York, USA

## Abstract

**Background:**

Genome Wide Association Studies represent powerful approaches that aim at disentangling the genetic and molecular mechanisms underlying complex traits. The usual "one-SNP-at-the-time" testing strategy cannot capture the multi-factorial nature of this kind of disorders. We propose a Hierarchical Naïve Bayes classification model for taking into account associations in SNPs data characterized by Linkage Disequilibrium. Validation shows that our model reaches classification performances superior to those obtained by the standard Naïve Bayes classifier for simulated and real datasets.

**Methods:**

In the Hierarchical Naïve Bayes implemented, the SNPs mapping to the same region of Linkage Disequilibrium are considered as "details" or "replicates" of the locus, each contributing to the overall effect of the region on the phenotype. A latent variable for each block, which models the "population" of correlated SNPs, can be then used to summarize the available information. The classification is thus performed relying on the latent variables conditional probability distributions and on the SNPs data available.

**Results:**

The developed methodology has been tested on simulated datasets, each composed by 300 cases, 300 controls and a variable number of SNPs. Our approach has been also applied to two real datasets on the genetic bases of Type 1 Diabetes and Type 2 Diabetes generated by the Wellcome Trust Case Control Consortium.

**Conclusions:**

The approach proposed in this paper, called Hierarchical Naïve Bayes, allows dealing with classification of examples for which genetic information of structurally correlated SNPs are available. It improves the Naïve Bayes performances by properly handling the within-loci variability.

## Background

In the last few years, the advent of massive genotyping technologies allowed researchers to define the individual genetic characteristics on a whole-genome scale. These advances boosted the diffusion of Genome Wide Association Studies (GWASs) and transformed them from expensive instruments of investigation into relatively affordable, popular and powerful research tools. For this reason, they have been extensively applied to the study of the most prevalent disorders.

As a matter of fact, most of the common diseases (e.g. diabetes mellitus, obesity, arterial hypertension, etc.) belong to the category of complex traits [1] which expression results from the additive contribution of a large spectrum of environmental determinants (exposure to external factors), behavioural factors (diet, life-style, smoke,...) and genetic variants (point mutations, single nucleotide polymorphisms - SNPs, large scale structural variations) [2]. Moreover, complex interactions among genetic variants, environmental factors and external influences are supposed to modulate not only the expression of the disease, but also the effectiveness of pharmacological treatments [3,4]. In this context, the identification of the molecular mechanisms underlying a certain disease could help researchers in forecasting the individual-level probability of developing specific disorders and thus in defining personalized pharmacological interventions. GWASs seem thus an interesting approach to cope with such issues by deepening the insight about the contribution of the genetic make-up of an individual to the probability of developing a certain disease or trait [2].

To date, from the statistical viewpoint, the main limitations to the full exploitation of the GWAS results are mostly represented by the lack of appropriate multivariate tools, which can replace the usual univariate testing strategies, commonly used during for the discovery phase of a GWAS. In standard univariate analyses, rules for defining statistically significant associations are usually based on the application of over-conservative significance thresholds, imposed to minimize the probability of false positive associations. The main drawback of these approaches is that they tend to discard potentially informative signals, resumed by genetic loci characterized by small effects on the trait [5].

In this context, multivariate models could overcome the limitations of the usual "one-SNP-at-a-time" testing strategies, offering the possibility of exploring and integrating the huge amount of information deriving both from whole genome screenings and from clinical/phenotypic measurements.

Beside logistic regression (LR), which represents the most common approach for building multivariate models from SNPs data [6], several standard and alternative machine learning approaches such Naïve Bayes (NB), Support Vectors Machines (SVM), Random Forests (RF), Least Absolute Shrinkage and Selection Operator (LASSO) and model-averaged Naïve Bayes (MANB) have been proposed and applied for dealing with GWAS data. NB represents a machine-learning method that has been used for over 50 years in biomedical informatics [7]. NB is computationally inexpensive and it has often been shown to reach optimal classification performances, even when compared to much more advanced and complex methods [8]. However, NB loses accuracy in presence of large amounts of attributes to be analyzed, since it tends to make predictions with posterior probabilities close to 0 and 1 [9]. SVMs are one of the most popular classifiers in the field of machine learning and achieves state-of-the-art accuracy in many computational biology applications [10]. Thanks to their performances, they have been applied recently in the context of GWAS [11,12]. Classification and Regression Trees (CART) represent machine learning algorithms that allow for the identification of predictive stratifications and functional interactions within data [13]. In the context of CART family of algorithms, RFs [14] allow analysing complex discrete traits using dense genetic information deriving from large sets of markers. In this context RFs are widely employed to the analysis of candidate genes association studies and GWAS for human binary traits [15]. Further, alternative approaches such logistic and Bayesian LASSO have been recently proposed and successfully applied for performing multivariate features selection in a genome-wide context [16-18], offering an appealing alternative to the standard univariate SNPs ranking and selection strategies.

Recently, Lee *et al. *[19] and Yang *et al. *[5] proposed two multivariate approaches based on the simultaneous fitting of a genome wide set of SNPs. In particular, Yang *et al. *[5] showed that about 45% of variance of the human height could be explained by considering simultaneously a whole - genome set of SNPs instead of focusing on a small fraction of highly significant hits. In a Bayesian framework, Wei *et al. *[20] proposed a model-averaged Naïve Bayes (MANB) to predict late onset Alzheimer's disease using about 310,000 polymorphic markers. These observations suggest that the genetic signature of an individual is represented by the information contained in its whole genome sequence more than in candidate loci.

Multivariate models, however, can be hardly learned from GWASs data due to the so-called "*small-n large-p problem"*: the number of variables in the model, i.e. the genotype loci, is much larger than the number of available individuals. This may cause major problems in model selection and model parameters fitting, instability and overfitting.

Bayesian methods, and in particular Bayesian Hierarchical Models (BHMs), represent a promising framework for deriving information from large sets of variables by exploiting available prior knowledge.

In our paper, we will exploit the capability of such models to use the knowledge about the correlation structure of such variables. Chromosome regions, represented by sequences of nearby SNPs, are often characterized by strong pairwise correlation, making the information available redundant and thus difficult to be analyzed. Hierarchical models (multilevel models) provide a way of pooling the information of correlated variables without assuming that they can be modelled as a unique variable [21]. Data coming from the same population are split in homogeneous subgroups, to which individual-level parameters are associated. The link/correlation among different individual parameters is expressed by population level parameters - or hyper-parameters. In this way it is possible to take into account for both within-group heterogeneity (thanks to the presence of individual level parameters) and between-groups variability (thanks to the presence of the population parameters).

BHMs have been already applied in a variety of biomedical contexts. They have been proposed as a fundamental tool to analyze next generation genomics data [22]. Moreover, Demichelis *et al. *applied such methods to tissue microarray data coming from tumor biopsies [21].

**Figure 1:**
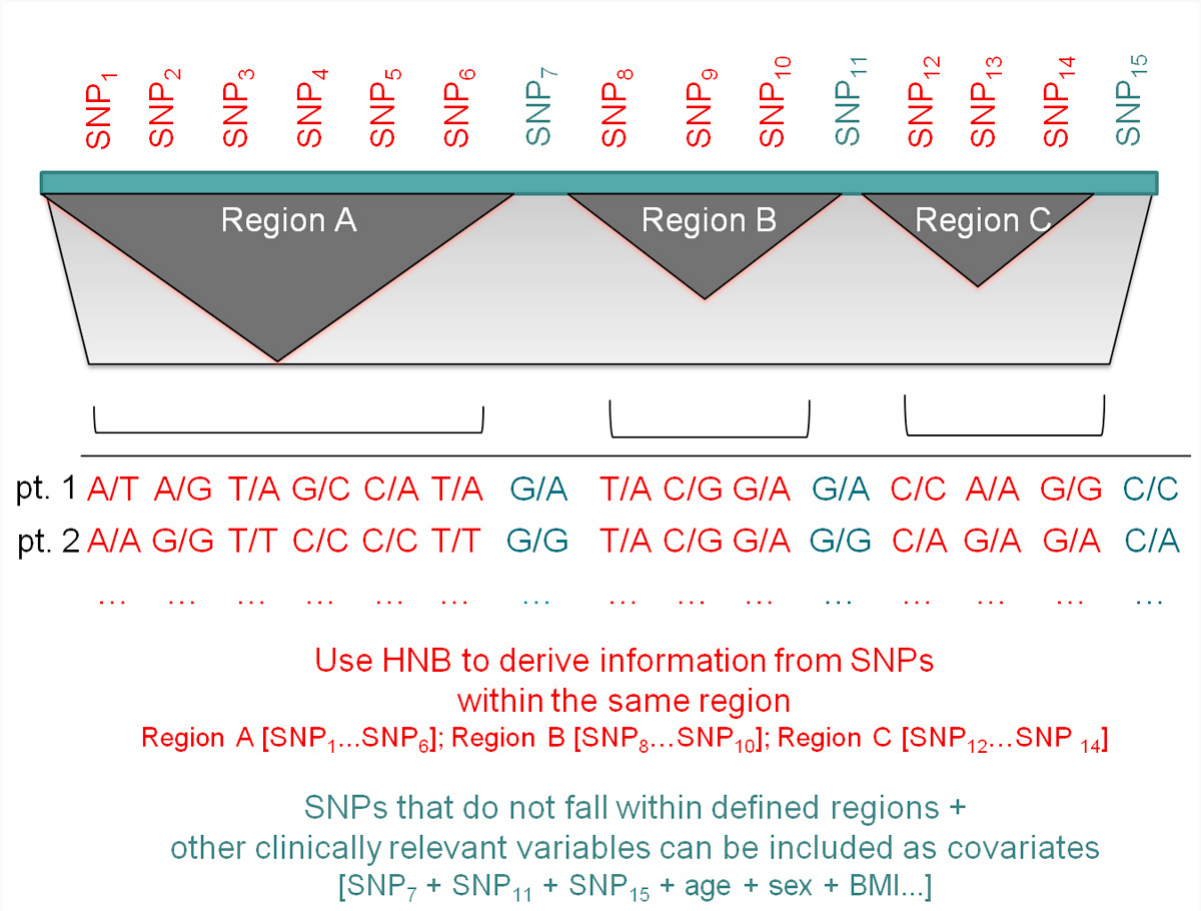
**Graphical representation of a genome region**.

In the context of GWASs, we propose a Hierarchical Naïve Bayes (HNB) classification model that allows capturing the uncertainty of the information deriving from a set of genetic markers that are functionally/structurally correlated and to use this information to classify new examples. SNPs that do not fall within such regions as well as clinically relevant variables (e.g.: gender, smoke, therapies, candidate markers) can be also included in the model (Figure 1).

The following sections describe the main methodological aspects of the algorithm implemented as well as the results obtained on both simulated datasets and two real GWASs on the genetic bases of Type 1 Diabetes (T1D) and Type 2 Diabetes (T2D) by the Wellcome Trust Case Control Consortium (WTCCC) [23].

## Methods

The Hierarchical Naïve Bayes classifier (HBN) is an extension of the well-known Naïve Bayes classifiers (NB). NB assumes that, given a class variable *C *that we aim at predicting (say disease yes/disease no) on the basis of a set of *n_f _*features *X *= {*x*_1_,..., *x_nf_*}, the posterior probability of the class given the data *P*(*C*|*X*) is proportional to the product of the prior probability of the class and the conditional probability, $P\left(X|C\right)\;=\;\prod_{f\;=\;1}^{n_{f}}P\left(X_{f}|C\right)$, i.e. that the features are independent among each other given the class. NB is a simple and robust classifier, which may be conveniently used also in the context of large number of features, due to its strong bias.

**Figure 2:**
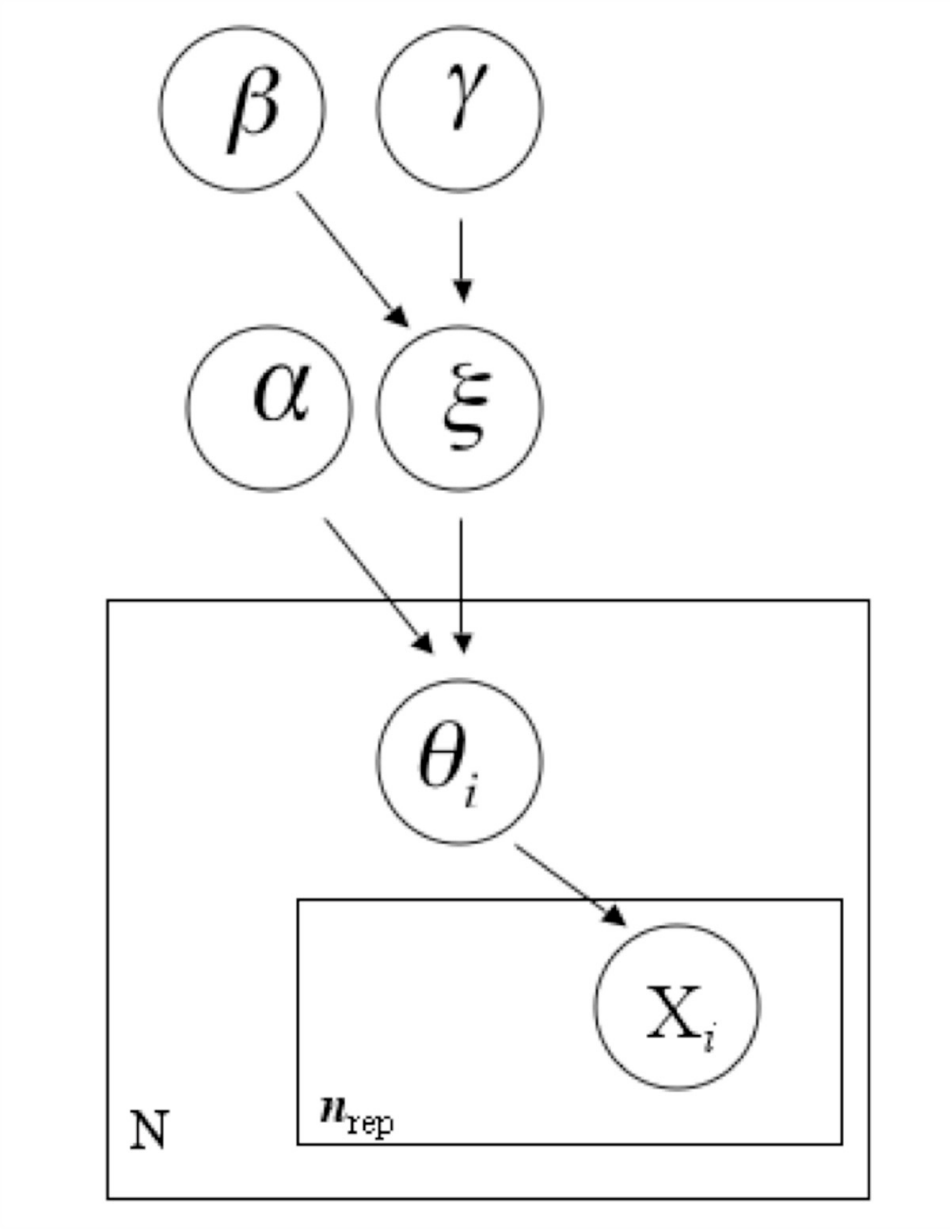
**The hierarchical structure of the data represented with plates notation**.

HBN assumes that the measurements are stochastic variables with a hierarchical structure in terms of their probability distributions. We suppose that we can collect a number *n_rep _*of observations, or replicates on each example, and that an example belongs to one of a set of given classes. Let us suppose that is a stochastic variable representing the replicates, whose probability distribution is dependent on a vector of parameters *θ*, which corresponds to the single example, and may represent, for example, the mean and variance of the probability distribution of replicates; if we consider the *i*-th example, with *i *in 1,..., *N*, the probability distribution of the vector of the replicates is given by $p_{\left(X_{i}|\theta_{i}\right)}$, with, $X_{i}\;=\;\left\{x_{i1},\;.\;.\;.\;,\;x_{ij},\;.\;.\;.\;,\;x_{in_{rep}}\right\}$, while the probability distribution of the individual parameters is $p_{\left(\theta_{i}|{\xi_{c}}_{{}_{k}}\right)}$, where $\xi_{C_{k}}$is a set of population hyper-parameters that depends on the class *C_k _*in the set *C *= {*C_1_*,... *C_h_*} to which the example belongs, and is thus the same for all the examples of the same class. Figure 2 shows the representation of the problems though a graphical model with plates [21].

In a Bayesian framework, the classification step is therefore performed by finding the class with the highest posterior probability distribution. Thanks to the conditional independence assumptions of the hierarchical model described above, we can write $P_{\left(C_{k}|X\right)}\propto P_{\left(X|\xi_{C_{k}}\right)}P_{\left(\xi_{C_{k}}|C_{k}\right)}P_{\left(C_{k}\right)}$. Since the population parameters $\xi_{C_{k}}$ are determined by the knowledge of the class *C_k _*with probability one, the equation can be simplified as $P_{\left(C_{k}|X\right)}\propto P_{\left(X|\xi_{C_{k}}\right)}P_{\left(C_{k}\right)}$. The posterior is thus dependent on the so-called marginal likelihood,$P_{\left(X|\xi_{C_{k}}\right)}$, which can be calculated by integrating out the vector of parameters *θ*.

Many replicates are available for each example. The examples are characterized by an individual vector of parameters *θ*; moreover, the examples belonging to the same class have a common set of parameters *ξ*.

(1) $$P(X|C_{k},\xi )=\int_{\Omega_{\theta }}P_{\left(X|C_{k},\theta \right)}P_{\left(\theta |C_{k},\xi \right)}d\theta$$

where **Ω***_θ _*is the support of *θ*.

The learning problem will therefore consist in estimating the population parameters $\xi_{C_{k}}$ for each class, while the classification problem is mainly related to the calculation of the marginal likelihood. To deal with multivariate problems, we resort to the Naïve Bayes algorithm (NB), which assumes that each attribute is conditionally independent from the others given the class.

(2) $$P\left(X|C_{k}\right)=\overset{n_{f}}{\underset{f=1}{\prod }}P\left(X_{f}|C_{k}\right)$$

From the computational viewpoint, this will allow us to compute separately the marginal likelihood for each variable to perform classification and to learn a collection of independent univariate models. In the following we will show how HNB deals with the classification and learning problems when the variables are discrete with multinomial distribution.

### Hierarchical Naïve Bayes for discrete variables

In a SNPs based case-control GWAS, the individual-level information is represented by genotype configurations (aa/aA/AA). For sake of readability we have omitted the dependence of the vectors to the class *k*. We assume that the vector of the occurrences (counts) of the *i-*th example is $X_{i}=\left\{x_{i1},\;\ldots \;,\;x_{ij},\;\ldots \;,\;x_{i_{S}}\right\}$, where $x_{i_{j}}$is the number of occurrences of the *j-th *discrete value, or state, of the *i-th *example and *S *is the number of states of the variable *x*. The number of replicates of each example is given by $n_{rep_{i}}=\sum_{j}^{S}x_{ij}$.

We also assume that the relationship between the data *X_i _*and the example parameters *θ_i _*is expressed by a multinomial distribution:

(3) $$X_{i}\;\sim Multin\left(n_{rep_{i}},\;\theta_{i1},\;\ldots ,\;\theta_{ij},\;\ldots ,\;\theta_{iS}\right)$$

Therefore *θ_i _*is an *S*-dimensional vector, where *θ_ij _*represents the probability of the occurrence of the *j-th *event in the example *i*. The parameters *θ_i_*, for *i *= *1, 2*,..., $N_{C_{k}}$, are characterized by the same prior Dirichlet distribution:

(4) $$\theta_{i}\;\sim \;Dirichlet\left(\alpha \xi_{1},\;\alpha \xi_{2},\;\ldots ,\;\alpha \xi_{S}\right)$$

with probability density:

(5) $$P\left(\theta_{i}|\alpha ,\;\xi \right)=\frac{\Gamma \left(\alpha \right)}{\prod_{j=1}^{s}\Gamma \left(\alpha \xi_{j}\right)}\;\overset{s}{\underset{j=1}{\prod }}\theta_{ij}^{\alpha \xi_{j}-1}$$

where 0 <*α *< ∞, *ξ_j _*< 1 ∀*j *= 1,..., *S *and $\sum_{j=1}^{s}\xi j\;=\;1$. Following the hierarchical model reported in the previous section, the individual example parameters *θ_i_*, are independent from each other given *ξ *= {*ξ_1_*,...,*ξ_S_*} and *α*. In the following we will assume that the parameter *α *will be fixed, and it will be thus treated as a design parameters of the algorithm. α represents the prior assumption on the degree of similarity of all examples belonging to the same class. A proper setting of the parameter *α *allows finding a compromise between a pooling strategy, where all replicates are assumed to belong to the same example and a full hierarchical strategy where all examples are assumed to be different.

### Classification

As described in the previous section, the classification problem requires the computation of the marginal likelihood (1). We assume that an estimate of the population parameters *ξ *is available and that *α, β *and *γ *are known. Given an example with counts distributed on different states *X *= {*x_1_*,..., *x_S_*}, where $n_{rep}=\;\sum_{j=1}^{s}xj$, we must compute:

(6) $$P(X|C_{k},\xi )=\int_{\Omega_{\theta }}P_{\left(X|\theta \right)}P_{\left(\theta |\xi_{C_{k}}\right)}d\theta$$

where *θ *= {*θ_1_*,..., *θ_S_*} is the vector of the individual example parameters, with $\sum_{j=1}^{s}\theta j=1$ and Ω*_θ _*the support of *θ*. This integral can be solved by noting that it contains the product of a Multinomial and a Dirichlet distribution.

The marginal likelihood can be thus computed as:

(7) $$P\left(X|C_{k},\xi \right)=\frac{n_{rep}!\;\Gamma \left(\sum_{j}\alpha \xi_{j}\right)}{\Gamma \left(\sum_{j}\left(x_{j}+\alpha \xi_{j}\right)\right)}\;\underset{j}{\prod }\;\frac{\Gamma \left(x_{j}+\alpha \xi_{j}\right)}{x_{j}!\;\Gamma \left(\alpha \xi_{j}\right)}$$

The NB approach allows to exploit this equation for each variable in the problem at hand, and then to apply the equation (2) to perform the classification. The marginal likelihood however requires the estimate of the population parameters *ξ *from the data.

### Learning with collapsing

The task of learning the population parameters can be performed by resorting to approximated techniques. Herein we will describe a strategy previously presented by [24] and [25].

We suppose that a data set *X *= {*X*_1_,..., *X_N_*} is available for each class where *X_i _*= {*x*_*i*1_,..., *x_is_*} and *N *is the number of examples within each class (the number of examples can differ between the classes). Such vector is transformed into a new vector X*where the *i*-th element $X_{i}^{*}=\left\{\tau_{i}x_{i1},\;\ldots ,\;\tau_{i}x_{ij},\;\ldots ,\;\tau_{i}x_{is}\right\}$ with:

(8) $$\tau_{i}=\frac{1+\alpha }{n_{rep_{i}}+\alpha }$$

*τ_i _*is a suitable weight that allows to take into account the prior assumptions on the heterogeneity of the example belonging to the class. The hierarchical model is then collapsed into a new model, where the vector of the measurements $X_{i}^{*}$ is assumed to have a multinomial distribution with parameters *ξ *and.$\tau_{i}x_{in_{rep_{i}}}$.

Such assumption can be justified by the calculation of the first and second moment of *P*_(X*|*ξ*) _which is computed by approximating the distribution of the parameters *θ *given *ξ *with its average value [25].

The Maximum Likelihood (ML) estimate of the parameters *ξ *can be thus obtained for each state of the discrete variable as:

(9) $$\bar{\xi_{J}}=\frac{\sum_{i=1}^{N}\tau_{i}x_{ij}}{\sum_{i=1}^{N}\tau_{i}n_{rep_{i}}}$$

Within this framework we can also provide a Bayesian estimate of the population parameters *ξ*. We assume that *ξ *is a stochastic vector with a Dirichlet prior distribution: *ξ *~ *Dirichlet*(*β_γ1_*,...., *β_γS_*), where 0 <*β *< ∞, *γ_j _*< 1 ∀ *j *= 1,..., *S *and $\sum_{j=1}^{s}\gamma_{j}=1$.

After collapsing, we may derive the posterior distribution of *ξ *is still a Dirichlet with expected value of the probability of the *j-th *state of the discrete variable:

(10) $$\bar{\xi_{J}}=\frac{\sum_{i=1}^{N}\tau_{i}x_{ij}+\beta \gamma_{j}}{\sum_{i=1}^{N}\tau_{i}n_{rep_{i}}+\beta }$$

In this setting, the parameter vector *γ *and *β *assume the same meaning of the parameters usually specified in the Bayesian learning strategies applied in many Machine Learning algorithms. In particular, if we assume *γ *= 1/*S *and *β *= 1 we obtain an estimate which is close to the Laplace estimate, while different choices of *γ *and *β *lead to estimates which are similar to the m-estimate, where *β *plays the role of *m*.

### Building the model

The HBN machinery can be conveniently exploited to build a multivariate model for SNPs coming from a GWAS. In presence of regions in which non-random association of alleles at two or more loci or Linkage Disequilibrium (LD) is observed [26], a new variable *X *is generated, and all the SNPs belonging to the same block are considered as replicate of the same variable (see Figure 1). On the contrary, if the SNPs are not in LD, they are treated as independent variables in equation (2). For this reason, the model needs a convenient pre-processing step, in which blocks of SNPs characterized by LD are identified and the variables extracted.

**Figure 3:**
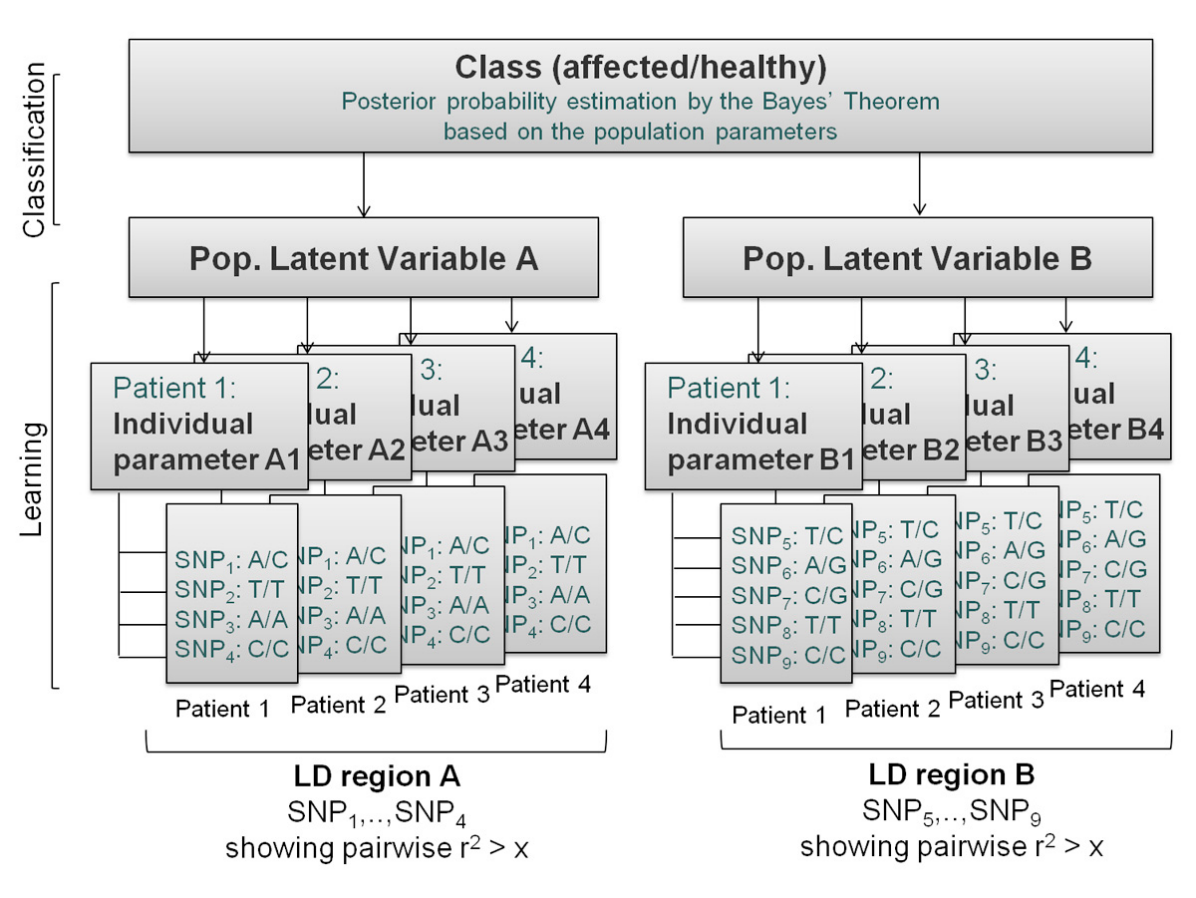
**The hierarchical structure of the data represented with the plates notation using SNPs data**.

Figure 3 reports a graphical representation of how SNPs data can be mapped using the plates notation. According to this representation, each individual is characterized by a vector (or individual parameter *θ*) reporting the genotypes corresponding to set of SNPs mapping to the same LD region. The set of individual parameters are then employed to estimate a latent variable *ξ: *each latent variable resumes the individual level information deriving from a different LD region. Thus, the complete set of latent variables (along with potentially informative covariates) is used in turns to estimate the probability of being affected or healthy by the Bayes' theorem.

## Results

### Datasets simulation

A total number of 9 independent datasets each composed by 300 cases, 300 controls and approximately 34,000 SNPs (representing the whole chromosome 22) have been simulated by the by the Hapgen software [23], according to the patterns of LD that characterize the HapMap CEU b36 reference population (http://hapmap.ncbi.nlm.nih.gov/). Three simulation scenarios have been evaluated, by imposing different genotype relative risk for causative loci:

• *Scenario 1*: heterozygote relative risk = 1.5, homozygote relative risk = 3.0

• *Scenario 2*: heterozygote relative risk = 2.0, homozygote relative risk = 4.0

• *Scenario 3*: heterozygote relative risk = 3.0, homozygote relative risk = 6.0

Three simulated datasets have been generated according to each scenario, by imposing Minor Allele Frequency (MAF) ≥ 0.05.

### Experimental datasets

The experimental case control datasets were represented by two genome-wide scans on T1D and T2D generated by the WTCCC consortium [23]. Individual-level genotypes determination has been performed with the Affymetrix GeneChip 500 K Mapping Array Set (http://www.affymetrix.com), which comprises 500,568 SNPs, while genotypes have been estimated from raw intensity signals by the Chiamo software tool [23].

Genotyped samples underwent a preliminary phase of data quality control (QC) which comprised the removal of cases and controls showing: i) missing data fraction > 3%;

ii) heterozygote genotypes fraction > 0.3 *OR *heterozygote genotypes fraction < 0.225;

iii) discordances or lack in terms of phenotype vs. laboratory information; iv) not-European ancestry; v) 1^st^/2^nd ^degree relatives; vi) duplicated samples. Analogously, SNPs QC consisted in removing markers characterized by: i) study-wise missing data proportion > 5% *OR *study-wise minor allele frequency < 5% *AND *study-wise missing data proportion > 1%; ii) statistically significant deviations from the Hardy-Weinberg Equilibrium within controls (p-HWE < 5.7 × 10^-7^); iii) 1 df Trend Test/2 df General test p-value < 5.7 × 10^-7 ^comparing allele and genotype frequencies between control groups; iv) bad clustering quality.

For a more detailed description of samples selection, genotyping procedures and quality control filters applied, the reader may refer to [23].

*T1D dataset*. The final dataset was composed by 1,963 patients affected by T1D, 1,458 control individuals from the UK Blood Service and 458,868 autosomal SNPs (mapping to chromosomes 1- 22) passing the quality control procedures.

*T2D dataset*. The final dataset was composed by 1,924 patients affected by T2D, 1,458 control individuals from the UK Blood Service and 458,868 autosomal SNPs (mapping to chromosomes 1- 22) passing the quality control procedures.

### Data pre-processing

Both simulated and experimental datasets underwent a preliminary phase of features selection and variables filtering aimed at i) reducing the space of the hypotheses to be tested and ii) isolating chromosome regions characterized by strong LD.

The main steps of the datasets preparation are reported below:

1. The whole datasets have been split randomly into screening (representing 70% of the whole dataset) and replication sets (the remaining 30% of the whole dataset). The sampling procedure has been performed with stratification, so that each fold was represented by the same proportion of cases and controls.

2. On each screening set:

a. Selected the top 500 most significant markers, based on the results from univariate Pearson *χ2 *tests with 2 degrees of freedom (df), comparing genotypes distributions between cases and controls.

b. Define chromosome regions characterized by the presence of nearby SNPs showing pairwise r^2 ^≥ *x*, where *x *represents arbitrary cut-off values corresponding to r^2 ^= 0.6 (SNPs in moderate-to-strong LD) and 0.8 (SNPs in strong LD) respectively.

i. Group markers localized within the same LD -block and build latent-variables.

ii. Use the remaining SNPs falling outside the LD-blocks as covariates.

c. Split the whole screening set into 10 folds of equal sample size and characterized by cases/controls ratio = 1 according to the 10 Folds Cross Validation procedure (10 Folds CV) [27].

3. Apply the LD-based SNPs grouping schema learnt on the screening set to the corresponding replication set.

Both screening and replication sets have been employed for evaluating the generalization performances obtained by the HNB algorithm and to compare them with those obtained by the standard NB classifier on the same datasets.

### Results from simulated datasets

The HNB algorithm has been validated on simulated datasets, which underwent the pre-processing phases described in the previous sections.

Descriptive analyses of the simulated datasets revealed that the number of blocks to be analyzed increased proportionally to the stringency of the r^2 ^imposed for defining regions of correlation, while the median number of SNPs characterizing each block decreased. This is due to the fact that SNPs linked by strong correlation (r^2 ^≥ 8), are generally confined to small and fragmented regions due to structural recombination events. Table 1 resumes the characteristics of the nine simulated datasets.

**Table 1:** Characteristics of the simulated datasets.

			**LD thr.: r**^**2 **^**≥ .0.60**			LD thr.: r^2 ^≥ 0.80	
			
sim	GRR	B	SNPs/B	**r**^**2**^	B	SNPs/B	**r**^**2**^
1	1.5/3.0	43	5.0 [6.50]	0.94 [0.13]	63	3 [5.50]	0.97 [0.06]
2	1.5/3.0	36	6.5 [11.50]	0.95 [0.08]	55	4 [6.00]	0.98 [0.05]
3	1.5/3.0	58	3.5 [5.00]	0.97 [0.07]	76	3 [3.00]	0.98 [0.06]

4	2.0/4.0	24	8.5 [29.50]	0.97 [0.07]	67	4 [4.00]	0.98 [0.06]
5	2.0/4.0	34	4.5 [14.00]	0.95 [0.17]	61	3 [6.00]	0.98 [0.09]
6	2.0/4.0	39	5.0 [6.50]	0.97 [0.19]	70	4 [3.75]	0.99 [0.05]

7	3.0/6.0	22	9.0 [28.25]	0.96 [0.07]	49	5 [6.00]	0.98 [0.07]
8	3.0/6.0	45	5.0 [10.00]	0.98 [0.10]	80	3 [3.00]	0.98 [0.06]
9	3.0/6.0	34	8.5 [14.50]	0.93 [0.11]	72	3 [4.00]	0.96 [0.09]

GRR, heterozygote/homozygote Genotype Relative Risk (GRR); B, number of blocks; SNPs/B, median number [Interquartile Range (IQR)] of SNPs within each block; r^2^, median [Interquartile Range (IQR)] pairwise r^2 ^within each block. The described parameters are reported for blocks defined using thresholds of LD corresponding to r^2 ^≥ 0.6 and 0.8 respectively.

The generalization performances of the two algorithms have been evaluated by comparing the Classification Accuracy (CA) and the Area Under the Curve (AUC) of the two models estimated by 10 Folds CV procedures and by testing the models learnt on single screening set on the corresponding independent replication set [27,28]. Results are reported in Table 2 and show that the HNB reaches higher or equal generalization performances with respect to the standard NB when chromosome regions characterized by SNPs showing moderate-to-strong (r^2 ^> 0.6) or strong (r^2 ^> 0.8) pairwise LD are analyzed.

**Table 2:** Results from the analysis of simulated datasets

				10 Folds CV		Independent Test	
					
sim	GRR	**LD thr**.	Model	CA	AUC	CA	AUC
1	1.5/3.0	r^2 ^≥ 0.6.	HNB	0.85 [0.81-0.87]	0.92 [0.91-0.95]	0.64	0.66
			NB	0.80 [0.78-0.82]	0.90 [0.89-0.90]	0.69	0.70
		
		r^2 ^≥ 0.8.	HNB	0.85 [0.81-0.89]	0.93 [0.90-0.95]	0.63	0.68
			NB	0.80 [0.78-0.82]	0.90 [0.89-0.90]	0.69	0.70

2	1.5/3.0	r^2 ^≥ 0.6.	HNB	0.87 [0.83-0.93]	0.94 [0.89-0.98]	0.63	0.68
			NB	0.83 [0.80-0.83]	0.87 [0.84-0.90]	0.59	0.63
		
		r^2 ^≥ 0.8.	HNB	0.85 [0.80-0.87]	0.92 [0.88-0.94]	0.65	0.70
			NB	0.83 [0.80-0.83]	0.87 [0.84-0.90]	0.59	0.63

3	1.5/3.0	r^2 ^≥ 0.6	HNB	0.73 [0.70-0.77]	0.82 [0.76-0.85]	0.65	0.72
			NB	0.78 [0.69-0.80]	0.86 [0.77-0.94]	0.68	0.75
		
		r^2 ^≥ 0.8	HNB	0.77 [0.70-0.80]	0.85 [0.80-0.88]	0.71	0.75
			NB	0.78 [0.69-0.80]	0.86 [0.77-0.94]	0.68	0.75

4	2.0/4.0	r^2 ^≥ 0.6	HNB	0.78 [0.72-0.84]	0.85 [0.80-0.89]	0.74	0.80
			NB	0.72 [0.64-0.81]	0.76 [0.72-0.86]	0.71	0.75
		
		r^2 ^≥ 0.8	HNB	0.72 [0.64-0.81]	0.77 [0.71-0.88]	0.70	0.76
			NB	0.72 [0.64-0.81]	0.76 [0.72-0.86]	0.71	0.75

5	2.0/4.0	r^2 ^≥ 0.6	HNB	0.82 [0.77-0.83]	0.89 [0.83-0.92]	0.73	0.80
			NB	0.78 [0.73-0.80]	0.84 [0.77-0.85]	0.76	0.83
		
		r^2 ^≥ 0.8	HNB	0.82 [0.78-0.83]	0.88 [0.84-0.90]	0.76	0.86
			NB	0.78 [0.73-0.80]	0.84 [0.77-0.85]	0.76	0.83

6	2.0/4.0	r^2 ^≥ 0.6	HNB	0.77 [0.73-0.80]	0.85 [0.83-0.87]	0.71	0.79
			NB	0.75 [0.68-0.77]	0.80 [0.76-0.82]	0.66	0.71
		
		r^2 ^≥ 0.8	HNB	0.73 [0.67-0.77]	0.80 [0.79-0.82]	0.65	0.72
			NB	0.75 [0.68-0.77]	0.79 [0.76-0.82]	0.66	0.71

7	3.0/6.0	r^2 ^≥ 0.6	HNB	0.83 [0.81-0.83]	0.91 [0.87-0.93]	0.76	0.84
			NB	0.80 [0.77-0.83]	0.85 [0.83-0.88]	0.81	0.87
		
		r^2 ^≥ 0.8	HNB	0.83 [0.80-0.86]	0.94 [0.93-0.94]	0.82	0.91
			NB	0.80 [0.77-0.83]	0.85 [0.83-0.88]	0.81	0.87

8	3.0/6.0	r^2 ^≥ 0.6	HNB	0.83 [0.78-0.87]	0.91 [0.89-0.94]	0.78	0.83
			NB	0.82 [0.80-0.86]	0.87 [0.82-0.94]	0.81	0.85
		
		r^2 ^≥ 0.8	HNB	0.82 [0.77-0.86]	0.90 [0.85-0.94]	0.78	0.86
			NB	0.82 [0.78-0.86]	0.87 [0.82-0.94]	0.81	0.85

9	3.0/6.0	r^2 ^≥ 0.6	HNB	0.92 [0.87-0.93]	0.96 [0.94-0.98]	0.86	0.92
			NB	0.83 [0.83-0.87]	0.92 [0.92-0.95]	0.84	0.86
		
		r^2 ^≥ 0.8	HNB	0.87 [0.87-0.92]	0.96 [0.93-0.97]	0.89	0.92
			NB	0.83 [0.83-0.87]	0.92 [0.92-0.95]	0.84	0.86

CA, Median Classification Accuracy and 25% - 75% of the distribution; AUC, Median Area Under the Curve and 25% - 75% of the distribution. The 25% - 75% of the distribution are reported for results deriving from 10 Folds CV.

Majority Classifier CA and AUC for 10 Folds CV and Independent test sets: 0.50

No significant variations in terms of CA and AUC have been observed as function of the different genotype relative risks imposed for data simulations (p > 0.05), thus CA and AUC estimated from different simulations have been pooled and used for evaluating the differences in terms of classification performances between HNB and NB.

Results show that the median CA and AUC obtained by the HNB over the single results are higher to those reached by the standard NB for both LD thresholds that have been evaluated. The one-tailed Wilcoxon signed rank test [29] has been used for testing the hypotheses that the CA and AUC obtained by the HNB were significantly higher than those estimated by the standard NB and by the majority classifier [30].

Results from the Wilcoxon signed rank test showed that:

• The distribution of the AUC values estimated by the HNB over the complete set of simulations was significantly higher than the corresponding distribution of AUC estimated by the standard NB when r^2 ^≥ 0.8 was imposed as threshold for defining LD-regions (AUC from 10 Folds CV: p < 0.05; AUC from independent replication set: p < 0.05).

• The HNB algorithm reached CA and AUC estimates significantly higher than those obtained by the majority classifier:

○ by comparing the distribution of CA and AUC obtained by the HNB with those generated by the majority classifier on the corresponding folds (maj. CA = 0.50, maj. AUC = 0.50) for each screening set according to both LD thresholds (p < 0.01);

○ by comparing the distribution of CA and AUC estimated by the HNB over the 9 independent test sets with the corresponding distribution of CA and AUC obtained by the majority classifier (maj. CA = 0.50, maj. AUC = 0.50) on the corresponding test set according to both LD thresholds (p < 0.01).

### Hierarchical Naïve Bayes for Type 1 and Type 2 Diabetes prediction

The HNB algorithm has been evaluated on two real genome-wide datasets aimed at identifying the genetic bases of T1D and T2D respectively. The analyzed datasets have been generated by the WTCCC [23] and they are publicly available. The final datasets were each composed by 1,400 cases and 1,400 controls sampled randomly from the complete set of individuals passing the quality control filters as reported in the previous section. Thus, each final dataset has been split into a fist set of 2,100 individuals (1,050 cases and 1,050 controls) representing the screening cohort, while the replication set was composed by the remaining 350 cases and 350 controls. The preliminary phases of features selection and LD-regions definition (using r^2 ^≥ 0.8 as threshold) have been performed as reported in methods section, SNPs that did not fall within conserved regions have been used as covariates.

The generalization performances of the proposed approach and of the NB have been estimated by i) 10 Folds CV performed on the each screening set and ii) by learning the models on the whole screening set and then testing the CA and AUC on the two corresponding replication cohorts.

Results are reported in Table 3 and confirm that the HNB algorithm is able to reach the highest generalization performances on both datasets, according to both 10 Folds CV and by testing the model learnt on the whole screening set on the corresponding independent replication cohort. Further, results from the Wilcoxon Signed Rank test evidenced that the distribution of CA and AUC obtained by the HNB by 10 Folds CV was significantly higher than the corresponding distributions obtained by the majority classifier on the same folds (p < 0.05).

**Table 3:** Results obtained on the T1D and T2D datasets

		10 Folds CV		Independent Test	
			
Study	Model	CA	AUC	CA	AUC
T1D	HNB	0.70 [0.67-0.73]	0.80 [0.78-0.82]	0.71	0.79
	NB	0.70 [0.67-0.72]	0.79 [0.76-0.81]	0.68	0.78
	
T2D	HNB	0.83 [0.81-0.85]	0.92 [0.89-0.93]	0.57	0.57
	NB	0.81 [0.80-0.84]	0.90 [0.89-0.92]	0.55	0.56

CA, Median Classification Accuracy and 25% - 75% of the distribution; AUC, Median Area Under the Curve and 25% - 75% of the distribution. The 25% - 75% of the distribution are reported for results deriving from 10 Folds CV. The described parameters are reported for blocks defined using thresholds of LD corresponding to r^2 ^≥ 0.8

Majority Classifier CA and AUC for 10 Folds CV and Independent test sets: 0.50.

## Discussion

The approach proposed, called Hierarchical Naïve Bayes, represents an innovative strategy aimed at exploiting correlated information from genome wide datasets. The human genome is typically characterized by local patterns of strong LD that define blocks of SNPs showing low recombination rates. In this scenario, the HNB represents a suitable way of deriving genetic information with respect to standard multivariate models, since it is able to take into account for structural correlations existing between markers. These characteristics allow HNB to overcome the limitations of the standard NB algorithm, which over-simplistic assumptions of independence between attributes are rarely respected in the context of GWAS data. The results obtained by the HNB on both simulated and real datasets show that the proposed approach is able to achieve classification performances that are generally higher or equal to those obtained by multivariate models based on standard NB. In particular, the HNB represents a suitable alternative to the standard NB when analyzing genome regions characterized by strong LD, a typical condition in which the assumptions of independency between variables of the HNB are dramatically violated.

To be noted, even if the results obtained by the 10 Folds CV procedures are prone to overfitting for both simulated and real datasets, since the preliminary filtering phase heavily exploits the screening set for features selection and blocks determination, the results obtained on the replication sets are free from these limitations. These observations confirm how taking into account for structural correlation between markers offers substantial gain in terms of generalization capability with respect to the standard NB approach that does not consider the human genome structure.

Many research groups used the publicly available WTCCC datasets and private case/control cohorts on T1D and T2D for testing the predictive performances of several machine learning algorithms. As an example, Wei *et al*., explored an approach based on SVM for building risk models using SNPs data and tested their approach on different case/control datasets on T1D [11]. The authors reported AUC ranging from 0.86 to 0.89 by 5 Folds CV, using different SNPs inclusion thresholds on the WTCCC cohort, while AUC corresponding to 0.84 and 0.83 by training the algorithm on WTCCC data and testing the performances on CHOP/Montreal-T1D and GoKinD-T1D datasets respectively, representing independent cohorts of cases and controls. When the algorithm was trained on the CHOP/Montreal-T1D and tested on the WTCCC and GoKinD-T1D data, the algorithm reached comparable AUC estimates, corresponding to 0.84 and 0.82 respectively. Roshan *et al. *[31] studied the number of causal variants and associated regions identified by top SNPs in rankings given by the 1 df chi-squared statistic, SVM and RF on real datasets on T1D from the WTCCC and GoKinD studies. SVM achieved the highest AUC of 0.83 with 21 SNPs followed by random forest and chi-square AUCs of 0.81 each with 29 and 17 SNPs, respectively. Clayton [32] discussed the impact of including interaction terms for predicting the probability of T1D and reported AUC estimated corresponding to 0.74 using pairwise interaction terms in logistic regression and 0.73 when no interaction were considered. These observations suggest how interaction between SNPs does not add substantial additional information to the correct classification of T1D subjects.

Lower CA and AUC estimates are generally obtained from the T2D datasets. As an example, van Hoek *et al. *investigated 18 polymorphisms from recent GWAS on T2D by logistic and Cox regression models in the Rotterdam Study cohort, reaching AUC corresponding to 0.60 [33]. Hyo-Jeong Ban *et al. *[12] analyzed a Korean population of T2D patients and controls, reporting CA corresponding to 0.65 using a combination of 14 SNPs in 12 genes mapping to T2D related pathways by using the radial basis function (RBF)-kernel SVM.

The performances obtained by the HNB on the independent test sets are generally comparable to those reported by other research groups for both T1D and T2D reported in this section. However, a direct comparison of the performances obtained by the HNB on the real datasets with those obtained by other previously published approaches on the same WTCCC cohorts can be hardly interpreted due to differences in terms of sample size of the control population (the analyzed dataset does not include the 1958 British Birth Cohort of controls, generated by the WTCCC and commonly used as reference population along with the UK Blood Service cohort). Further, the lack of covariates regarding T1D and T2D cases and controls (e.g., BMI, smoking history,.., etc.) limited the possibility to integrate genetic and clinical information, a key step for a deeper comprehension of complex trait diseases. Thus, the availability of GWAS datasets complete of detailed phenotype and clinical information will allow testing the HNB in a more realistic scenario. Beside these considerations, the proposed approach can be further improved to take into account also functional correlations, by using, for example, the Tree Augmented Naïve Bayes (TAN) approach on the latent variables, thus combining the two strategies [34].

## Competing interests

The authors declare that they have no competing interests.

## Authors' contributions

AM carried out the molecular genetic studies, performed the statistical analysis and drafted the paper. NB carried out software tools development and integrations, participated in study design and drafted the manuscript. RB conceived the study, participated in its design and coordination and helped to draft the manuscript. All authors read and approved the final manuscript.
